# ABCG36/PEN3/PDR8 Is an Exporter of the Auxin Precursor, Indole-3-Butyric Acid, and Involved in Auxin-Controlled Development

**DOI:** 10.3389/fpls.2019.00899

**Published:** 2019-07-09

**Authors:** Bibek Aryal, John Huynh, Jerôme Schneuwly, Alexandra Siffert, Jie Liu, Santiago Alejandro, Jutta Ludwig-Müller, Enrico Martinoia, Markus Geisler

**Affiliations:** ^1^Department of Biology, University of Fribourg, Fribourg, Switzerland; ^2^Institut für Botanik, Technische Universität Dresden, Dresden, Germany; ^3^Institute for Plant and Microbial Biology, Zurich, Switzerland

**Keywords:** IBA, IAA, ABCG, ABC transporter, PDR, PEN3, auxin, plant development

## Abstract

The PDR-type ABCG transporter, ABCG36/PDR8/PEN3, is thought to be implicated in the export of a few structurally unrelated substrates, including the auxin precursor, indole-3-butyric acid (IBA), although a clear-cut proof of transport is lacking. An outward facing, lateral root (LR) location for ABCG36 fuelled speculations that it might secrete IBA into the rhizosphere. Here, we provide strong evidence that ABCG36 catalyzes the export of IBA – but not of indole-3-acetic acid – through the plasma membrane. ABCG36 seems to function redundantly with the closely related isoform ABCG37/PDR9/PIS1 in a negative control of rootward IBA transport in roots, which might be dampened by concerted, lateral IBA export. Analyses of single and double mutant phenotypes suggest that both ABCG36 and ABCG37 function cooperatively in auxin-controlled plant development. Both seem to possess a dual function in the control of auxin homeostasis in the root tip and long-range transport in the mature root correlating with non-polar and polar expression profiles in the LR cap and epidermis, respectively.

## Introduction

Initially, IBA was described as a synthetic auxin that elicited auxin-like effects such as root initiation and was thus used for plant propagation in a process called rooting (reviewed in Frick and Strader, 2018). It has now been established that IBA is an endogenous compound in a variety of plant species examined (Frick and Strader, 2018) Its existence, however, in the model plant *Arabidopsis thaliana* has been questioned (Novak et al., 2012). Currently, it appears that this might simply be a question of extraction and detection methods (Frick and Strader, 2018; Matern et al., 2019). However, until now, it is unclear if IBA acts as an auxin by itself or if it acts strictly via IAA, the major auxin, for which it functions as a precursor (Ludwig-Muller, 2007; Strader and Bartel, 2008; Schlicht et al., 2013). Conversion of IBA to IAA is a peroxisome-dependent reaction (Zolman et al., 2008) and peroxisomal import is thought to occur by PEROXISOMAL TRANSPORTER1/COMATOSE/ABCD1 (PXA1/CTS/ABCD1) belonging to the ABCD family of ABC transporters (Zolman et al., 2001; Footitt et al., 2002; Hooks et al., 2007).

Indole-3-butyric acid uptake is a saturable process (Rashotte et al., 2003), suggesting that IBA uptake into plant cells is mediated by unidentified IBA uptake carriers (Frick and Strader, 2018; Matern et al., 2019). IBA export is thought to be catalyzed by members of the PDR transporters belonging to the ABCG family of ABC transporters that is limited to plants and fungi (Strader and Bartel, 2009; Kang et al., 2010; Kretzschmar et al., 2012). Interestingly, members of the PIN and ABCB families of IAA transporters do not appear to transport IBA (Ruzicka et al., 2010; Matern et al., 2019) indicating an independent evolution of auxin substrate specificities for distinct transporter classes. Plant PDRs have been assigned to the transport of substrates that are involved in several biotic and abiotic responses: for example, tobacco PDR1 was identified as an exporter of the antifungal terpenoid, sclareol (Jasinski et al., 2001), while PDR5 is induced by the defense hormone, jasmonic acid, and wounding and plays a role in herbicide resistance (Bienert et al., 2012). The PDR, Leaf Rust Resistance34 (Lr34), confers durable, race-specific resistance to multiple fungal pathogens in wheat (Krattinger et al., 2009), while *Petunia* PDR1 has been shown to export the hormone strigolactone in the context of arbuscular mycorrhizal establishment (Kretzschmar et al., 2012).

Mutant alleles of *PDR8/PEN3/ABCG36* (hereafter referred to as ABCG36) were first shown to display altered responses to diverse pathogens (Kobae et al., 2006; Stein et al., 2006), decreased extracellular accumulation of flagellin22-induced callose (Clay et al., 2009) and hyper-accumulation of flagellin22- or pathogen-elicited indole glucosinolate derivatives of the PEN2 pathway (Bednarek et al., 2009; Clay et al., 2009). A striking feature of ABCG36 is its focal accumulation at the site of leaf pathogen entry, where it is thought to export as yet unidentified defense compounds through the plasma membrane (PM) (Stein et al., 2006; Xin et al., 2013). Recently, the indole, 4-methoxyindol-3-yl methanol, was identified as a substrate for ABCG36 functioning in the induced deposition of callose by flagellin22 (Matern et al., 2019).

On the other hand, *abcg36* alleles were found to independently hyper-accumulate and to be hypersensitive to IBA (Strader and Bartel, 2009; Lu et al., 2015). In the root, ABCG36 is predominantly laterally localized at the outermost root PM domains (Langowski et al., 2010; Ruzicka et al., 2010). In these lateral domains, which were defined as the root-soil interface (Langowski et al., 2010; Ruzicka et al., 2010), ABCG36 was shown to co-localize widely with ABCG37 and was thus suggested to act redundantly in mediating root auxin homeostasis (Ruzicka et al., 2010). In agreement, both *ABCG36* and *ABCG37* were identified in chemical genetic screens for hypersensitivity toward auxinic compounds and auxin transport inhibitors, including IBA (Strader and Bartel, 2009; Ruzicka et al., 2010; Frick and Strader, 2018). Accumulation and reduced efflux from entire root tips were found for different *abcg36* alleles (Strader and Bartel, 2009). Finally, by heterologous expression, ABCG37/PDR9/PIS1 was shown to function as a transporter of IBA and 2.4-D but not of IAA (Ruzicka et al., 2010). In summary, genetic and biochemical approaches in Arabidopsis support the idea that IBA efflux from root cells is catalyzed by at least two PDRs of the ABCG family of ABC transporters, ABCG36/PDR8/PEN3 (Strader and Bartel, 2009; Lu et al., 2015) and ABCG37/PDR9/PIS1 (Strader et al., 2008; Ruzicka et al., 2010). IBA hypersensitivity phenotypes of mutants defective in these transporters suggest that IBA is a common substrate exported by both ABCG36 and ABCG37 (Strader and Bartel, 2009) but clear IBA transport data have so far only been provided for ABCG37 (Ruzicka et al., 2010).

Here we provide strong evidence that ABCG36 functions as a PM exporter of IBA that dampens polar (rootward) IBA transport in the root. Further, our data support a functional interplay with ABCG37 in auxin-controlled plant physiology.

## Materials and Methods

### Plant Material and Phenotypic Analyses

The following *Arabidopsis thaliana* lines in ecotype Columbia (Col Wt) were used: *abcg36-4 (pen3-4)* (Stein et al., 2006), *abcg37-2 (pdr9-2)* (Ito and Gray, 2006), *abcg36-4 abcg37-2 (pen3-4 pdr9-2)* (Ruzicka et al., 2010)*, 35S:ABCG37-GFP (35S:PIS1-GFP)* (Ruzicka et al., 2010)*, ABCG36:ABCG36-GFP (PEN3:PEN3-GFP)* (Stein et al., 2006). *DR5:GFP* (Ottenschlager et al., 2003), was crossed into *abcg36-4 and abcg37-2* and isogenic, homozygous lines for the transgene in the F3 generations were used for further analyses. *35S:ABCG36* Arabidopsis lines were constructed by transforming *35S:PDR8* (Kim et al., 2007) into the *abcg36-4*/*pen3-4* mutant.

Seedlings were generally grown on vertical plates containing 0.5 Murashige and Skoog media, 1% sucrose, and 0.75% phytoagar in the dark or at 16 h (long day) light per day. Developmental parameters, such as primary root lengths, lateral root (LR) number and root gravitropism, primary root and hypocotyl elongation were quantified from scanned plates using the Fiji plugin^1^ for ImageJ^2^. All experiments were performed at least in triplicate with 30 to 40 seedlings per experiment.

### Yeast Work

cDNA of ABCG36 was PCR-amplified and inserted into BamHI/ XhoI sites of pYES2NT/C-ABCG36. ABCG36 and ABCG37 were expressed from shuttle vectors pYES2NT/C-ABCG36, pNEV-ABCG37-HA (Ruzicka et al., 2010) in Wt strain JK93da (Hemenway and Heitman, 1996). Yeast IBA/2.4-D transport was performed as described (Kim et al., 2010).

### Confocal Laser Scanning Imaging

For confocal laser scanning microscopy work, a SP5 confocal laser microscope was used. Confocal settings were set to record the emission of GFP (excitation 488 nm, emission 500–550 nm) and FM4-64 (excitation 561 nm, emission 600–680 nm).

### Plant Auxin Transport

Simultaneous export of [ring-^3^H]-IBA (specific activity 25 Ci mmol^−1^; American Radiolabeled Chemicals, ART1112) with either [carboxyl-^14^C]-2.4-D (specific activity 50 mCi mmol^−1^; American Radiolabeled Chemicals, ARC0722) or [1-^14^C]-IAA (specific activity 55 mCi mmol^−1^; American Radiolabeled Chemicals, ARC1060) from Arabidopsis and *Nicotiana benthamiana* mesophyll protoplasts was analyzed as described (Henrichs et al., 2012). *N. benthamiana* mesophyll protoplasts were measured 4 days after *Agrobacterium*-mediated transfection of *35S:ABCG36/35S:PDR8* (Kim et al., 2007) or *35S:ABCG37-GFP* (Ruzicka et al., 2010). In short, after loading, external radioactivity was removed by separating protoplasts in a 50-30-5% Percoll gradient. Transport was initiated by incubation at 25°C and halted by silicon oil centrifugation. Exported radioactivity was determined by scintillation counting of aqueous phases and is presented as the relative efflux of the initial efflux (efflux prior to temperature incubation), which was set to zero. Relative export from protoplasts was calculated from exported radioactivity into the supernatant as follows: (radioactivity in the supernatant at time t = x min) – (radioactivity in the supernatant at time t = 0) ^∗^ (100%)/(radioactivity in the supernatant at t = 0 min); mean values are presented from four independent experiments. Rootward (acropetal) and shootward (basipetal) PAT in roots was measured as described in Lewis and Muday (2009).

### *In planta* Analysis of Auxin Contents and Responses

Endogenous free IBA and IAA were quantified from shoot and root segments of 9-days-after-germination (dag) light-grown (16 h) Arabidopsis seedlings by using gas chromatography-mass spectrometry (GC–MS) as described in Leljak-Levanic et al. (2010). Methylation was performed by adding equal sample amounts of a 1:10 diluted solution (in diethylether) of trimethylsilyldiazomethane solution (Sigma-Aldrich) for 30 min at room temperature. The mixture was then evaporated and resuspended in 50 μl of ethyl acetate for GC–MS analysis. Data are means of four independent lots of 30–50 seedlings each, and equivalent to ca. 100 mg root shoot material, respectively.

Homozygous generations of Arabidopsis *abcg36-4* and *abcg36-4 abcg37-2* expressing DR5:GFP were obtained by crossing with DR5:GFP lines (Ottenschlager et al., 2003). Seedlings were grown vertically for 5 (dag) and then for 4 h on 5 (M IBA plates and analyzed by confocal laser-scanning microscopy. DR5:GFP signals in the very root tip were analyzsed using the Fiji software^3^.

### Data Analysis

Data were statistically analyzed using Prism 7.0a (GraphPad Software, San Diego, CA, United States) and the R software package of the Comprehensive R Archive Network (CRAN)^4^.

## Results

### ABCG36 Functions as an Exporter of IBA

Recently, by heterologous expression in yeast and HeLa cells, ABCG37/PDR9/PIS1 was shown to function as a transporter of IBA, 2.4-D and NPA but not of IAA (Ruzicka et al., 2010). For different *abcg36* alleles, increased accumulation and reduced efflux from entire root tips were measured (Strader and Bartel, 2009) but no direct transport data, especially in the absence of plant-specific factors, was provided.

**FIGURE 1:**
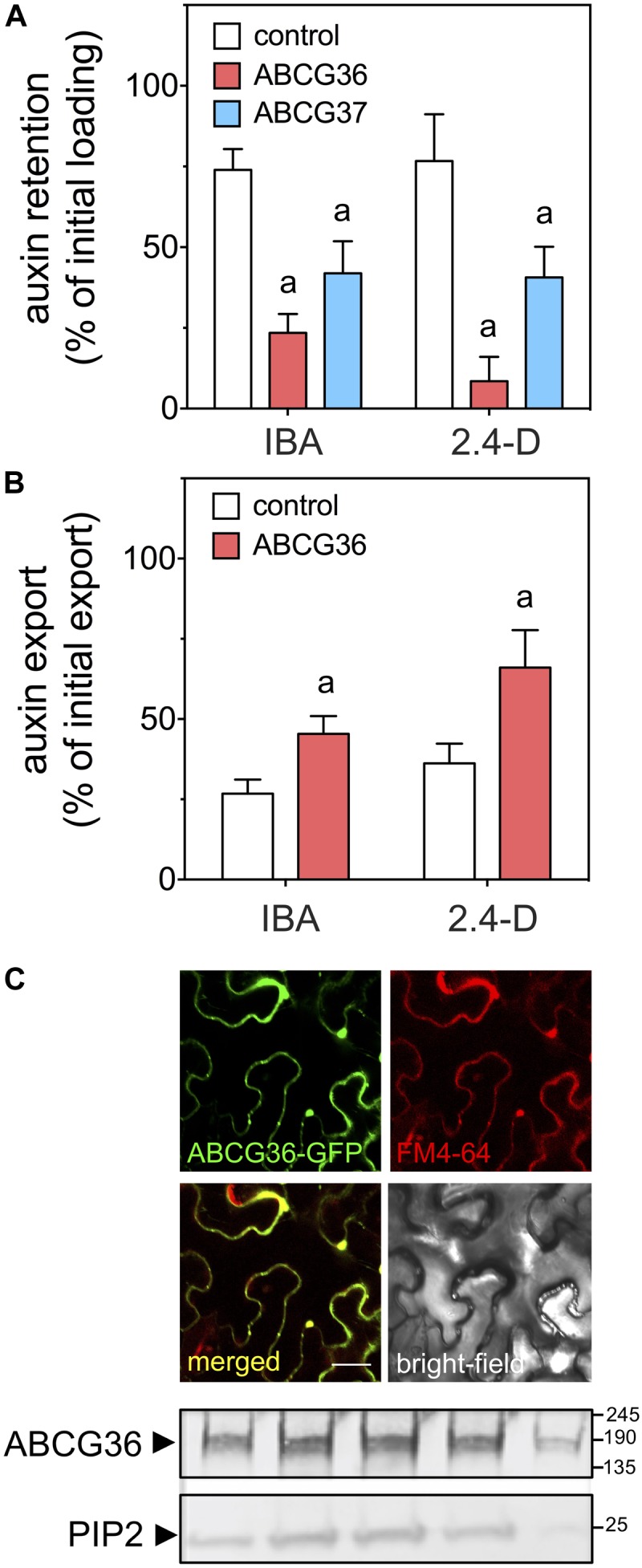
Heterologous expression of ABCG36 results in enhanced plasma membrane export of the native and synthetic auxins, IBA and 2.4-D. **(A)** Yeast expressing ABCG36 or ABCG37 retain significantly less IBA and 2.4-D indicating higher export. **(B)** Enhanced IBA and 2.4-D export from *Nicotiana benthamiana* protoplasts prepared after *Agrobacterium*-mediated leaf transfection with 35S:*ABCG36.* Significant differences (unpaired *t*-test with Welch’s correction, *p* < 0.05) to vector control are indicated by an ‘a’ (mean ± SE; *n* ≥ 4 transport experiments generated from independent yeast transformations or tobacco transfections). **(C)** ABCG36 is expressed on the plasma membrane revealed by confocal imaging of ABCG36-GFP (upper panel) and Western analyses of sucrose gradient fractions prepared from tobacco leaves transfected with *ABCG36:ABCG36-GFP.* Microsomes were separated by a linear 10–50% sucrose gradient and plasma membrane (PIP2)-positive fractions 9–13 (corresponding to 37–50% sucrose) were probed with anti-GFP (lower panel).

In order to demonstrate such a transport activity, we functionally expressed *ABCG36* in the heterologous, non-plant and plant systems, baker’s yeast and tobacco (*N. benthamiana*), respectively. ABCG36- and ABCG37-expressing yeast (the latter was used here as a positive control) revealed significantly reduced retention of IBA and 2.4-D, assayed in parallel by double isotope labeling (Figure 1A). Reduced retention argues for an export activity. *N. benthamiana* transfection resulted in expression of ABCG36 at the PM of epidermal cells as was shown by co-localization with FM-4-64 after short-term incubation of transfected leaves (Figure 1C). Confocal imaging was verified biochemically by demonstrating co-sedimentation of ABCG36-positive microsomal fractions with the PM marker, PIP2;1, using Western blotting after fractionation on a linear sucrose gradient. ABCG36 expression on the PM greatly enhanced IBA and 2.4-D export from prepared tobacco protoplasts (Figure 1B) as was shown before for ABCG37 (Ruzicka et al., 2010).

**FIGURE 2:**
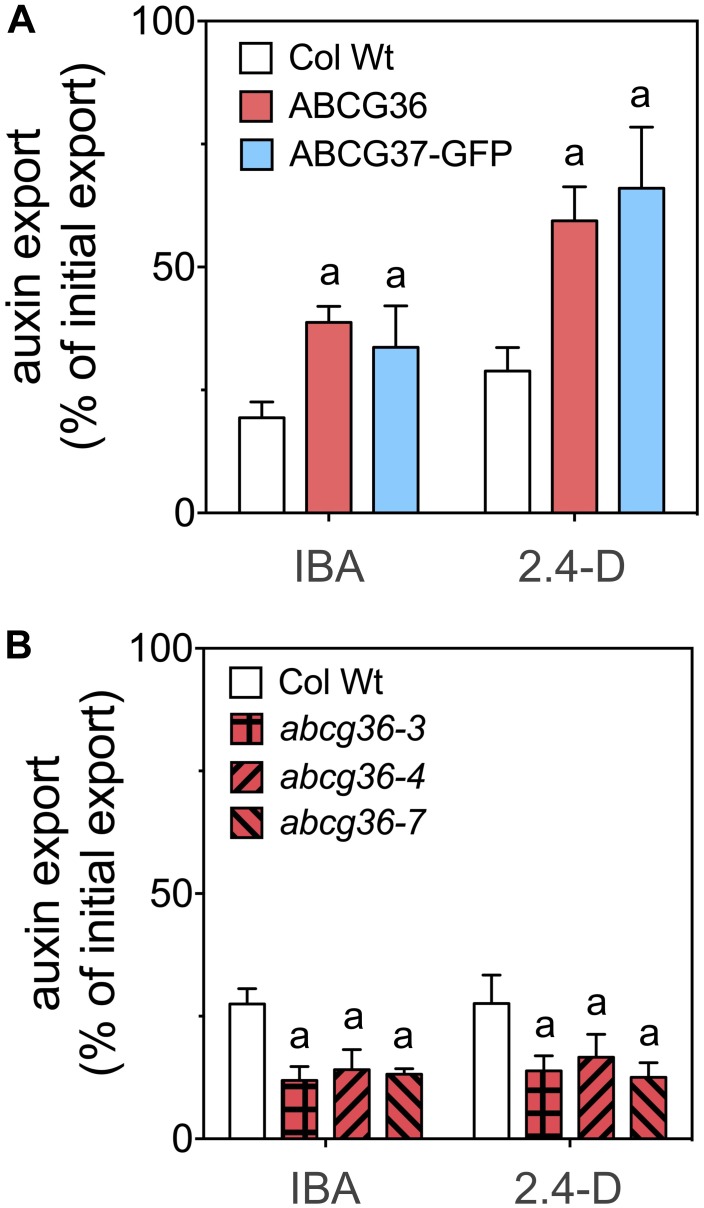
ABCG36 transports the native and synthetic auxins, IBA and 2.4-D, in Arabidopsis. **(A)** Enhanced IBA and 2.4-D efflux from leaf protoplasts prepared from *ABCG36* (*35S:ABCG36*) and *ABCG37* (*35S:ABCG37-GFP*) gain-of-function plants. **(B)** Reduced IBA and 2.4-D efflux from leaf protoplasts prepared from indicated *ABCG36* loss-of-function plants. Significant differences (unpaired *t*-test with Welch’s correction, *p* < 0.05) to Wt (Col Wt) are indicated by an ‘a’ (mean ± SE; *n* ≥ 4 transport experiments generated from independent protoplast preparations).

Next, we quantified IBA/2.4-D export from stable Arabidopsis lines over-expressing ABCG36 (Kim et al., 2007) and ABCG37-GFP (Ruzicka et al., 2010). Both lines exported significantly more IBA and 2.4-D (Figure 2A) but not IAA (Supplementary Figure 1) compared to the corresponding Wt. In agreement, T-DNA insertion, null alleles of *ABCG36*, *abcg36-3*, *abcg36-4*, *abcg36-7*, previously shown to express no ABCG36 protein (Lu et al., 2015), revealed significantly reduced IBA and 2.4-D export (Figure 2B). In summary, these data verify a PM export activity of ABCG36 for IBA but additionally indicate a high degree of substrate specificity as was already found for ABCG37 (Ruzicka et al., 2010).

### ABCG36 Functions in the Polar Distribution of IBA

**FIGURE 3:**
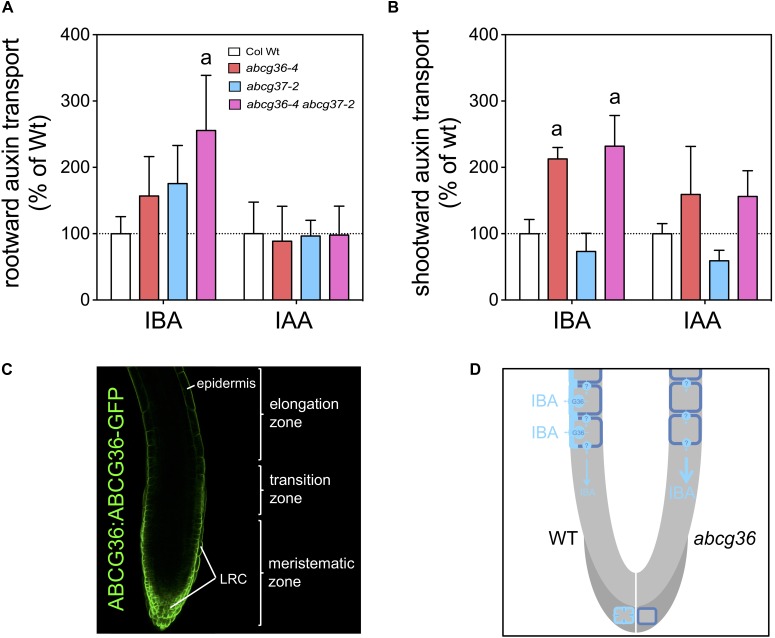
ABCG36 functions in rootward IBA transport in the Arabidopsis root. Rootward (**A**, acropteal) and shootward (**B**, basipetal) root transport of ^3^H-IBA and ^14^C-IAA assayed in parallel. Significant differences (unpaired *t*-test with Welch’s correction, *p* < 0.05) between WT and mutant alleles are indicated by an ‘a’ (mean ± SE; *n* ≥ 4 transport experiments). Both radiotracers were applied to root-shoot junctions and root tips by diffusion into plant tissues from agar beads functioning as source (Lewis and Muday, 2009); 5 mm segments from the source were used for quantification. **(C)** ABCG36-GFP is localized to the periphery of epidermal cells but reveals a non-polar expression in cells of the lateral root cap (LRC). Confocal image of a 5 dag root tip carrying *ABCG36:ABCG36* (*PEN3:PEN3-GFP*) in *abcg36-3/pen3-3* (Underwood and Somerville, 2017). **(D)** Putative model on the role of ABCG36 in polar transport of IBA. ABCG36 contributes to the rootward (acropetal) transport of IBA, which based on **(A,B)** seems to be shared by ABCG37 (not shown here). Lateral, outward-facing PM expression in the WT root epidermis (left panel) suggests lateral IBA excretion into the rhizosphere, taking IBA out of the polar IBA stream provided by so far uncharacterized apical-basal IBA transporters. Deletion of ABCG36 in the mutant (right panel) would enhance polar IBA transport, which is in line with our data. Additionally, ABCG36 seems to contribute to local auxin homeostasis in the very root tip correlating with a predominant non-polar expression in the LRC (dark gray). Absence of *ABCG36* might thus abolish IBA export resulting in DR5-GFP activation (Figure 4) and root hypersensitivity on IBA (Strader and Bartel, 2009).

Previous work has established that in roots both IBA and IAA both move with distinct polarities at similar transport rates (Rashotte et al., 2003). Inspired by the striking polar expression of ABCG36 and ABCG37 in the root epidermis (Ruzicka et al., 2010; Mao et al., 2016; see Figure 3C) and in order to demonstrate an involvement of ABCG36 in this process, we quantified polar IBA and IAA transport in 5 mm segments from the source. Both radiotracers were applied to root-shoot junctions and root tips by diffusion into plant tissues from agar beads functioning as source (Lewis and Muday, 2009). Results revealed to our surprise that rootward (acropetal) transport is enhanced in *abcg36, abcg37* and *abcg36 abcg37* mutant roots, although significantly differences were only found with the double mutant (Figure 3A). This effect is specific for IBA as it was not found with IAA assayed simultaneously. Shootward (basipetal) transport rates were likewise significantly enhanced in *abcg36* and *abcbg36 abcbg37* but not in *abcg37* roots, which might indicate a selective contribution of ABCG37 in this transport stream. Slightly reduced, although not significant, shootward transport in *abcg37* roots is in agreement with a recent report (Ruzicka et al., 2010), however, it is important to mention that application methods employed differ. While we here applied radiolabeled IAA by placing agar beads containing low amount of radiotracers next to the plant tissue (Lewis and Muday, 2009), Ruzicka et al. (2010) used direct nano-droplet application to the plant tissues, which might result in external passage of auxin-containing microfluids by capillary forces.

Enhanced rootward and shootward transport rates for *ABCG36/37* transporter loss-of-function mutants might seem counter-intuitive at first sight. In light of the strict lateral, outward-facing distribution for ABCG36 (Strader and Bartel, 2009, see Figure 3C) and ABCG37 (Ruzicka et al., 2010) in the region that is relevant for this transport measurement, a plausible explanation is that both transporters might function in lateral exclusion of IBA from the apical-basal transport stream. Loss-of-function would thus result in enhanced polar IBA transport (see Figure 3D). Altogether, this dataset further substantiates that ABCG36 like ABCG37 acts as an IBA exporter and is involved in the regulation of its polar distribution by a yet unidentified apical-basal transport machinery (Figure 3D).

### ABCG36 and ABCG37 Have an Impact on Auxin Signaling and Accumulation

**FIGURE 4:**
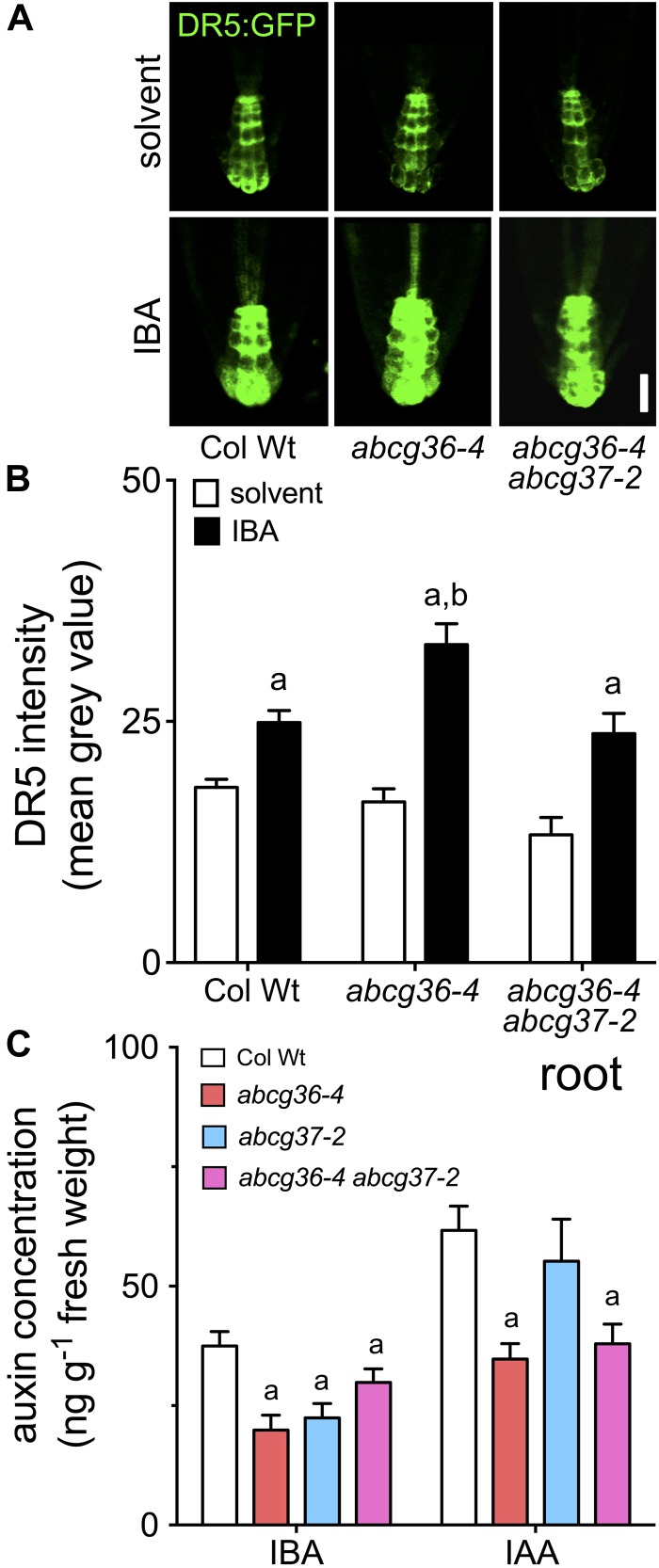
Auxin responses and quantification of free IBA and IAA in *abcg36*. Root auxin responses visualized by the auxin-responsive DR5 reporter in the root tip of 5 dag seedlings **(A)** grown on solvent (DMSO) or 5 μM IBA (**B,** quantification of fluorescence by image analysis of confocal sections). Significant differences (unpaired *t*-test with Welch’s correction, *p* < 0.05) to corresponding solvent controls are indicated by an ‘a,’ while differences between IBA-treated WT and mutant alleles are marked by a ‘b’ (mean ± SE; *n* ≥ 20 images). Bar: 20 μm. **(C)** Free IBA quantified by GC–MS is reduced in *abcg36/37* roots. Significant differences (unpaired *t*-test with Welch’s correction, *p* < 0.05) between WT and mutant alleles are indicated by ‘a’ (mean ± SE; *n* = 4). Shoot quantification is shown in Supplementary Figure 2.

Recently, hyper-sensitivity of *abcg37-1/pis1-1* roots to IBA was correlated with increased induction of the auxin (IAA) signaling reporter, DR5:GFP, known to be not activated by IBA itself (Ruzicka et al., 2010). Based on quantification of DR5:GFP expression in the root tip, auxin signaling was not found to be different in *abcg36-4* and *abcg36-4 abcg37-2* compared to WT on control media (Figure 4A,B), as shown for *abcg37-1/pis1-1* (Ruzicka et al., 2010). However, like *abcg37-1* (Ruzicka et al., 2010), application of IBA led to significantly higher reporter activation in the *abcg36-4* columella cells compared to the WT, and results in additional signals in the stele (Figure 4A,B). Interestingly, this effect was reverted in the *abcb36-4 abcg37-2* double mutant to Wt level arguing for compensating roles of both transporters in the columella cells (see section “Discussion”).

In order to provide more direct evidence for an involvement of ABCG36 (and ABCG37) in IBA/IAA homeostasis and to dissect IBA and IAA – but also ABCG36,37 – functionalities, we analyzed free IBA and IAA levels from entire root and shoot segments of Arabidopsis seedlings by GC–MS. Both roots and shoots of *abcb36* and *abcg37* single and double mutants contained significantly less IBA than the WT most likely caused by defects in apical-basal IBA distribution (Figure 4C and Supplementary Figure 2). Interestingly, IAA levels in *abcb36-4* and *abcb36-4 abcg37-2* roots – but not in those of *abcg37-2* – were also reduced, which is in line with enhanced polar transport rates for IBA (Figure 3). In summary, this data set uncovers that ABCG36 and ABCG37 have both an impact on root and shoot IBA and IAA homeostasis but that their individual impact is tissue-specific.

### ABCG36 Is Involved in Some – But Not All – Auxin-Controlled Developmental Programs in an Action That Is Partly Shared With ABCG37

Originally, the *ABCG36* allele, *abcg36-6/pdr8-115*, was isolated in a screen for mutants that were able to restore IBA but not IAA responsiveness to auxin signaling mutants (Strader and Bartel, 2009). In the following, individual mutant alleles of *ABCG36* were tested for their sensitivity toward IBA using root growth as read-out but different technical setups revealed mixed reports: while *abcg36-1/pen3-1*, *abcg36-2/pen3-2*, *abcg36-3-pen3-3*, *abcg36-4/pen3-4*, and *abcg36-6* were found to be hypersensitive (Ruzicka et al., 2010; Strader and Bartel, 2011; Lu et al., 2015), the point mutation allele, *abcg36-5*, was not (Lu et al., 2015). For the *abcg37-2/pdr9-2* allele, hypersensitivity toward IBA as well as a redundant function with ABCG36 were reported (Ruzicka et al., 2010).

**FIGURE 5:**
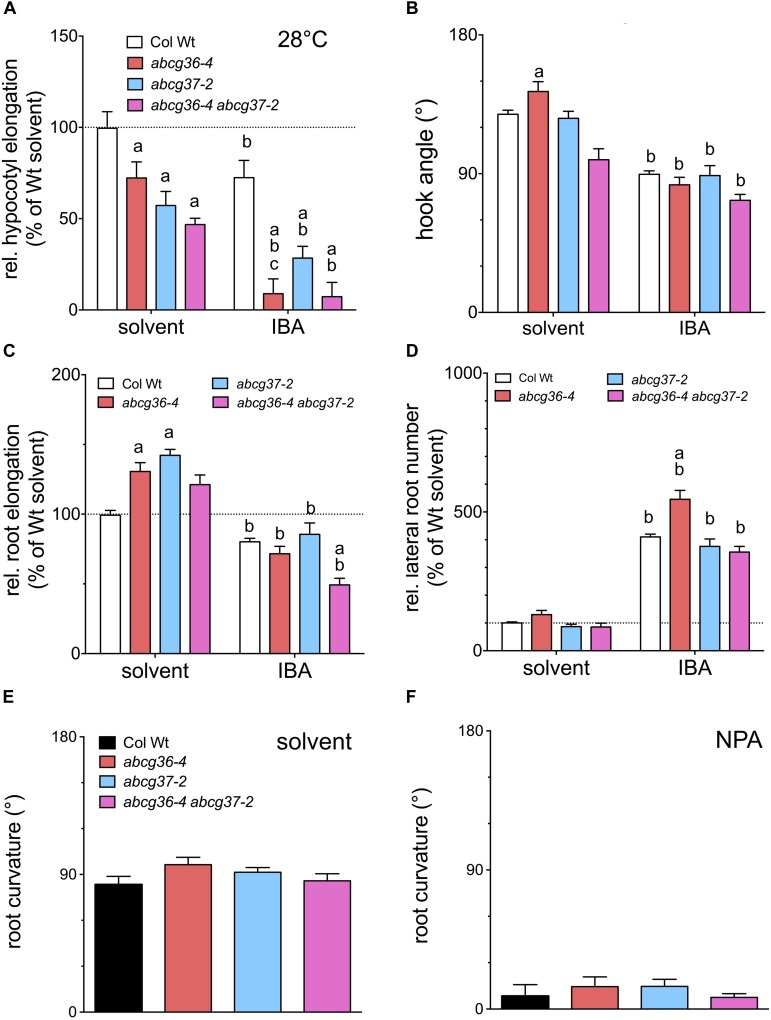
ABCG36 contributes to auxin-controlled plant development. **(A)** Hypocotyl elongation of 5 dag seedlings after transfer onto solvent control or IBA (5 μM) plates after 3 days at 28°C. 22°C control is shown in Supplementary Figure 3. **(B)** Hook opening angle of etiolated seedlings grown for 3 days in the dark and transferred onto solvent control or IBA (5 μM) plates in the dark after 4 days. **(C)** Root elongation of 5 dag seedlings after transfer onto solvent control or IBA (5 μM) plates after 24 h. **(D)** Quantification of emerged lateral roots of 5 dag seedlings after transfer onto solvent control or IBA (5 μM) plates after 7 days. Significant differences (unpaired *t*-test with Welch’s correction, *p* < 0.05) between corresponding WT and mutant alleles are marked with an ‘a,’ differences to corresponding WT solvent control with a ‘b’ (means ± SE; *n* = 4 sets of > 20 seedlings each). For root bending assays, 5 dag seedlings were transferred to solvent control **(E)** or NPA (10 μM) plates **(F)**, and root bending angles were judged after 12 h in the dark.

We therefore re-analyzed the performance of an established set of *ABCG36* and *ABCG37* single and double loss-of-function lines (Ruzicka et al., 2010) in respect to well-characterized, auxin-controlled growth programs. In order to be able to potentially dissect the role of individual ABCG-type transporters in IBA homeostasis, we also quantified seedling phenotypes after transfer to IBA plates. While hypocotyls at standard conditions (22°C) had no obvious phenotypes, growth at 28°C – known to cause a shift to thermomorphogenesis characterized by elongated hypocotyls most likely triggered by enhanced IAA (Gray et al., 1998; Gil and Park, 2019) – greatly reduced hypocotyl elongation in the order *abcg36* < *abcg37* < *abcg36 abcg37* in comparison to WT (Figure 5A). IBA enhanced this phenotype at 28°C but not at 22°C suggesting that increased conversion of IBA to IAA might be responsible for this effect. Analysis of hook opening in etiolated seedlings, another hallmark of auxin-regulated shoot development (Abbas et al., 2013), revealed that single *ABCG36* or *ABCG37* mutation did not significantly affect hook opening angles, while the *abcg36-4 abcg37-2* double mutant revealed an impaired hook opening angle (Figure 5B). IBA treatment generally inhibited hook opening compared to the solvent control but also ameliorated the differences between the mutant alleles.

Results for root elongation indicated that *abcg36-4* and *abcg37-2* roots elongate faster on solvent plates as was previously found (Strader and Bartel, 2009; Ruzicka et al., 2010), while this phenotype is lost in *abcg36-4 abcg37-2* (Figure 5C). As found for hook opening, IBA inhibited root elongation overall. As shown before *abcg36-4 abcg37-2* was hypersensitive in comparison to Wt (Ruzicka et al., 2010), while *abcg36-4* and *abcg37-2/pdr9-2* was not. The latter is in agreement with a former report (Ito and Gray, 2006) who likewise employed a transfer of seedlings onto IBA medium, while apparently, growth on IBA produces different results (Ruzicka et al., 2010). As a second developmental root parameter, we measured the number of emerged LRs. Again, it was found that *abcg36-4* roots produce slightly more LRs (cf Strader and Bartel, 2009). According to its known rooting potential, IBA increased LR numbers for all accessions tested, and the *abcg36-4* allele was clearly hyper-responsive toward IBA (Strader and Bartel, 2009), while *ABCG37* mutant combinations were not (Figure 5D). As a last physiological constraint, we measured root gravitropism for the same mutant set, which to our knowledge has not been analyzed in this respect. In contrast to most measured read-outs, all *ABCG36* and *ABCG37* loss-of-function alleles revealed Wt-like root bending (Figure 5E). Also, all mutant lines showed similar sensitivities toward the non-competitive auxin transport inhibitor, *N*-1-naphthylphtalamic acid (NPA; see Figure 5F), that efficiently blocks IAA-controlled root gravitropism (Bailly et al., 2008). The finding that *abcg* mutant roots bend like Wt on NPA might indicate that both transporters are not sensitive to NPA as was suggested before for IBA transport in general (Liu et al., 2012).

In summary, these data support an involvement of ABCG36 in most of the auxin-controlled developmental programs tested, such as hypocotyl and root elongation and hook opening. This action of ABCG36 is partly shared with ABCG37, as illustrated by hypocotyl elongation and hook opening. However, root gravitropism data suggest that ABCG36 (and ABCG37) is not involved in all auxin-controlled processes. Our analyses also revealed that IBA altered all four of the developmental read-outs tested. Amongst those, hypersensitivity of *ABCG36/37* mutant alleles to IBA (as for hypocotyl elongation, root elongation and LR development; Figure 5) further supports the involvement of ABCG36/37 in auxin-controlled development.

## Discussion

### ABCG36 Is an IBA Exporter Negatively Regulating Rootward IBA Transport

The current work enhances the portfolio of ABCG36 substrates by the auxin precursor, IBA. This has been established by heterologous expression in non-plant (yeast) and plant systems (tobacco; Figure 1). Recently, expression of ABCG37 in yeast resulted in mislocation to the ER leading to enhanced yeast loading (Ruzicka et al., 2010). Using an identical approach and even the same construct for ABCG37, we here report PM export for ABCG36 and ABCG37; discrepancies in localizations for ABCG37 in yeast between Ruzicka et al. (2010) and this study are currently not known. However, PM exports of IBA (and 2.4-D) were verified in tobacco and *in vivo* by analyses of Arabidopsis *ABCG36* gain- and loss-of-function mutant lines (Figure 1, 2).

It appears that ABCG36 and ABCG37 apparently possess a wider, but clearly delimited, specificity for a few overlapping but structurally unrelated substrates found to be typical for PDR-type ABC transporters (Kang et al., 2010; Borghi et al., 2015). However, it should be mentioned that the substrate sets described are not identical: ABCG37 but not ABCG36 was found to transport phenolic compounds, including coumarin (Fourcroy et al., 2014, 2016; Ziegler et al., 2017). Interestingly, in this substrate category, ABCG36 (like ABCG37) seems to be specific for a few auxinic compounds, such as IBA and 2.4-D, but both do not, for example, transport the major native auxin, IAA (Supplementary Figure 1), which only differs from IBA by a two-carbon chain. A future venue for this interesting finding might be offered by the *abcg36-5/pen3-5* allele that was reported to uncouple ABCG36 function in IBA-stimulated root growth, callose and a pathogen-inducible accumulation of salicylic acid from ABCG36 activity in extracellular defense (Lu et al., 2015).

Previous reports left us with a certain degree of uncertainty if IBA is indeed transported in a polar fashion (Rashotte et al., 2003; Ruzicka et al., 2010; Liu et al., 2012). While Rashotte et al. (2003) provided evidence for polar IBA transport in the shoot and roots without analyzing the radiotracer, Liu et al. (2012) clearly demonstrated polar IBA movement, distinct from that of IAA in the hypocotyl. In contrast, Ruzicka et al. (2010) demonstrated that already after 2 h most of the IBA applied to roots is metabolized into IAA. Our careful analysis of PAT in Arabidopsis roots revealed that ABCG36 functions redundantly with ABCG37 in rootward IBA (but not IAA) transport (Figure 3A). As discussed above, their role seems to lie in diminishing rootward IAA streams provided by so far unknown IBA transporters, by concerted, lateral IBA export out of the root (Figure 3D). Enhanced rootward, long-range IBA transport leads apparently to reduced concentrations of free IBA in *abcg36-4* and *abcg37-2*, measured here over entire roots. Although a final proof of lateral, epidermal ABCG36/37-mediated IBA export is lacking due to technical limitations, the overall concept is in agreement with increased accumulation and reduced efflux from *abcg36* root tips (Strader and Bartel, 2009).

At first view, ABCG36 (but not ABCG37) also seems to have a negative impact on shootward transport of IBA in the root (Figure 3B). However, shootward IAA transport profiles remarkably resemble those of IBA, although both ABCG transporters were shown not to transport IAA (Figure 3B; Ruzicka et al., 2010). Therefore, we cannot exclude that differences in shootward IAA transport might be caused by peroxisomal conversion of ring-labeled IBA to IAA during the elevated time-frame that is technically necessary for measuring this slow directionality (18 h for shootward vs. 3 h for rootward transport). Although the impact of IBA-to-IAA conversion in Arabidopsis roots has been discussed controversially (Ruzicka et al., 2010; Liu et al., 2012), we believe that polar root transport of IBA using radiotracers can only be safely quantified in a rootward direction.

In contrast to direct IBA/IAA quantifications of entire roots, signals of the columella, maximum IAA reporter, DR5:GFP, were unchanged in *abcg36-4* and *abcg36-4 abcg37-2* on solvent control, as found for *abcg37-2* (Ruzicka et al., 2010). In analogy, IBA significantly enhanced reporter expression compared to Wt in *abcg36-4* (Figure 4A,B), most likely by IBA to IAA conversion caused by lack of LRC export. In summary, these data support a dual function of ABCG36 in the polar (rootward) distribution of IBA in the root and local IBA/IAA homeostasis in the very root tip. These features correlate with the mainly polar (lateral) expression pattern in the mature root and a widely non-polar expression in the LR cap cells (see Figure 3D; Strader and Bartel, 2009; Ruzicka et al., 2010; Mao et al., 2016, 2017).

### ABCG36 and ABCG37 Are Partly Redundant

Widely overlapping substrate profiles and PM localizations between ABCG36 and ABCG37 imply but do not directly prove functional redundancy. A meta-analysis of our data supports the concept that ABCG36 and ABCG37 indeed function at least partly redundantly. This is confirmed by analyses of hook opening, of hypocotyl elongation at 28°C and of root elongation in the presence of exogenous IBA (Figure 5). Leaving uncertain or indirect observations, like shootward PAT of IBA or DR5-GFP reporter activation, aside, a few other read-outs do not support a common function in the same pathway. For example, LR development that is enhanced with IBA in *abcb36-4* (Figure 5D; Strader and Bartel, 2009) seems to be independent of ABCG37. This is surprising because LRs are thought to be initiated by a shoot-derived auxin maximum (Casimiro et al., 2003; Schlicht et al., 2013), and based on our data, shootward IBA root transport is apparently provided by both ABCG36 and ABCG37 (Figure 4, 5).

Interestingly, quantification of auxin responses, free IBA and root elongation in *abcg36 abcg37* roots (Figure 2), revealed a certain degree of compensation of individual single-mutant phenotypes. In light of an overall redundant function in IBA transport this is not easy to understand. An important finding in this respect might be that ABCG37 was found to be a frequent interactor of ABCG36 (Aryal and Geisler, unpublished). An appealing scenario is therefore that ABCG36 and ABCG37 itself functionally interact, which might explain both the discrepancies found during compensatory and redundant function. The impact of full-size ABC-ABC transporter interaction is currently still unclear but has been recently supported by uncovering the ABC transporter interactome in yeast (Snider et al., 2013).

### Analyses of *abcg36/37* Mutants Allowed to Partially Dissect the Roles of IBA and IAA Transport Pathways

The analyses presented here for *abcg36/37* mutants in respect to auxin-controlled growth processes allowed us to partially dissect the roles of IBA and IAA transport pathways and their involvement in either IBA or IAA-controlled plant development. As pointed out above, all of the growth parameters investigated were affected by IBA (Figure 5). More interestingly, *abcg36/37* mutants were hypersensitive to IBA in all four of the tested developmental read-outs, although to different degrees (Figure 5): while this effect seems to be specific for ABCG36 during hypocotyl elongation and LR initiation (Figure 5A,D), this functionality seems to be shared with ABCG37 during root elongation. A concerted action of ABCG36 with ABCG37 contributes to the polar (rootward) distribution of IBA in the plant root and is responsible for auxin-controlled development.

## Data Availability

All datasets generated for this study are included in the manuscript and/or the Supplementary Files.

## Author Contributions

MG and BA designed the research. BA, JS, JH, AS, JL-M, and JL performed the research. SA and EM provided unpublished material. BA, JS, JH, JL-M, and MG analyzed the data. MG wrote the manuscript.

## Conflict of Interest Statement

The authors declare that the research was conducted in the absence of any commercial or financial relationships that could be construed as a potential conflict of interest.

## Footnotes

1 https://imagej.net/Fiji

2 https://imagej.net

3 https://imagej.net/Fiji

4 http://cran.r-project.org

## Abbreviations

ABCB: ATP-binding cassette protein subfamily B
ABCG: ATP-binding cassette protein subfamily G
dag: day(s) after germination
dai: day(s) after infection
IAA: indole-3-acetic acid
IBA: indole-3-butyric acid
NPA: 1-*N*-naphthylphthalamic acid
PAT: polar auxin transport
PDR: pleiotropic drug resistance
PIN: pin-formed
WT: wild-type.
