# Supplementary material for: ABCG36/PEN3/PDR8 Is an Exporter of the Auxin Precursor, Indole-3-Butyric Acid, and Involved in Auxin-Controlled Development
Source: Front Plant Sci. 2019 Jul 9;10:899. doi: 10.3389/fpls.2019.00899 (PMC6629959; doi:10.3389/fpls.2019.00899)
Supplement: Supplementary file 1 [file Data_Sheet_1.PDF]

Supplementary Figures

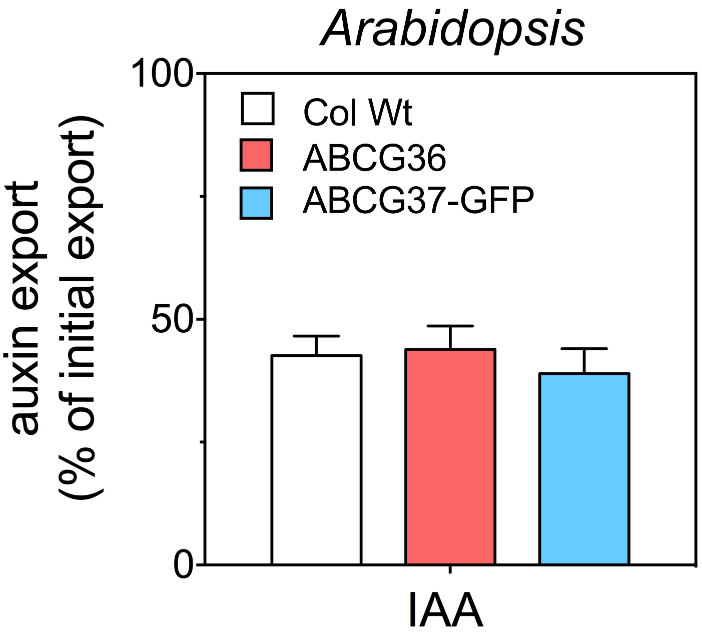

Supplementary Figure 1: Neither ABCG36 nor ABCG37 transport IAA.
Efflux of IAA from protoplasts prepared from stable Arabidopsis *ABCG36* and *ABCG37* gain-of-function (35S:ABCG36/ABCG37-GFP) lines. Differences (unpaired *t* test with Welch's correction, $p < 0.05$) between wild type and mutant alleles are not significant (mean ± SE; n ≥ 4 independent protoplast preparations).

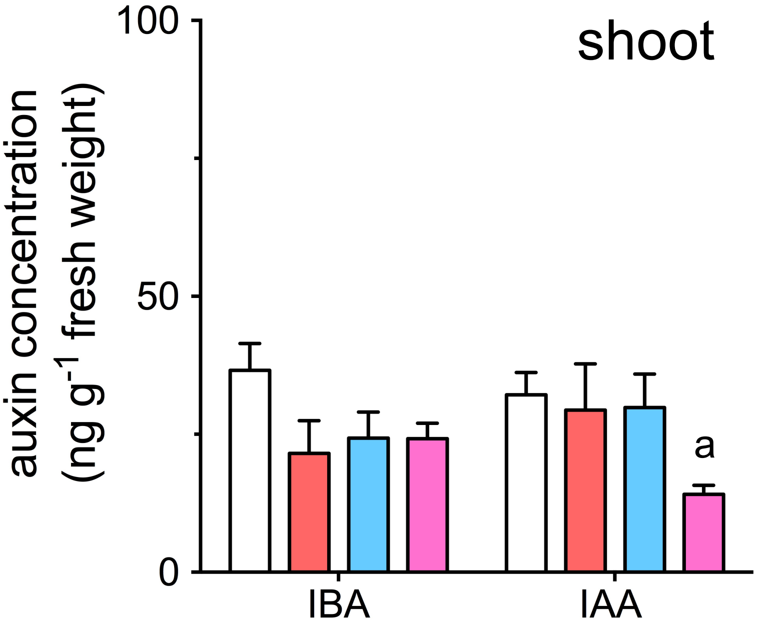

Supplementary Figure 2: Free IBA and IAA quantified by GC-MS is not reduced in *abcg36* shoots. Free IBA quantified by GC-MS is not reduced in *abcg36/37* roots. Significant differences (unpaired *t* test with Welch's correction, $p < 0.05$) between wild type and mutant alleles are indicated by 'a' (mean ± SE; n =4). Root quantification is shown in Fig. 4C.

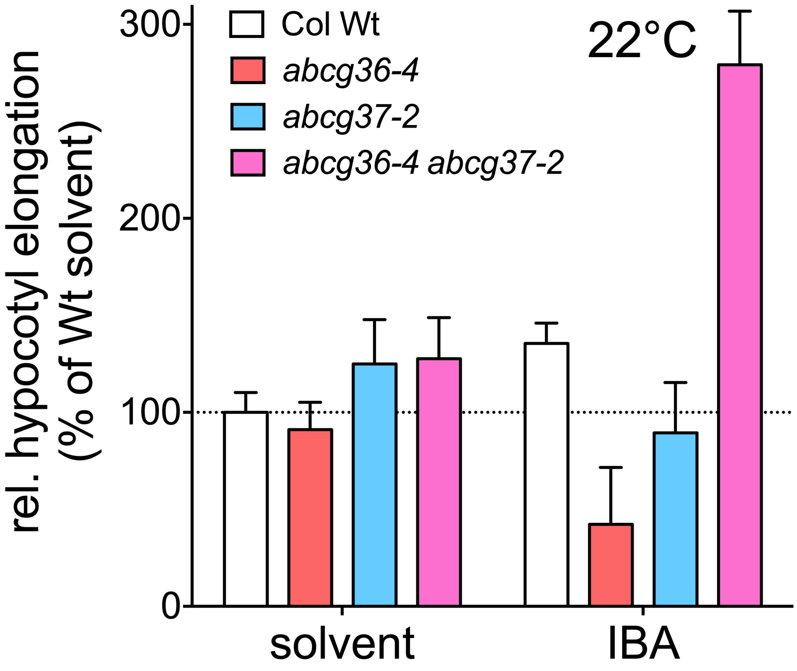

Supplementary Figure 3: ABCG36 and ABCG37 do not have a significant impact on hypocotyl elongation at 22°C.

For quantification of hypocotyl elongation, 5 dag seedlings were transferred to solvent control or IBA (5 µM) plates and relative hypocotyl elongation was judged after 3 days at 22°C. 28°C data are part of Fig. 5.

Significant differences (unpaired t test with Welch's correction, $p < 0.05$) between wild-type and mutant alleles with 'a' (means ± SE; n = 4 sets of 20 seedlings each).
